# 

**Published:** 1882-11

**Authors:** 


					﻿Vol. II.
NOVEMBER, 1882.
NO. 11.
HJHE
pOMCEOPATHlC pHY^lClAfl,
A MONTHLY JOURNAL OF MEDICAL SCIENCE.
“ Magna est'veriias, el prcet'alebit.” '
HL JL LEE, EL. HL,
EDITOR. .
CONTENTS:
A (See inside "of Cover.)
PHILADELPHIA:
No. 2109 CHESTNUT STREET.
r. O 3ST x> o 2ST.
ALFRED. .HEATH & CO,, Sous.Agents bob Great Britain,-
114 Ebury Street, Eaton Square.
Yearly Subscription, $2.50. invariably in advance.
Entered at the Philadelphia Post-Office as Second Class Mail Matter.
				

